# Phosphorylation‐mediated PI3K‐Art signalling pathway as a therapeutic mechanism in the hydrogen‐induced alleviation of brain injury in septic mice

**DOI:** 10.1111/jcmm.17568

**Published:** 2022-10-29

**Authors:** Yuanyuan Bai, Li Li, Beibei Dong, Wanjie Ma, Hongguang Chen, Yonghao Yu

**Affiliations:** ^1^ Department of Anesthesiology Tianjin Institute of Anesthesiology, General Hospital of Tianjin Medical University Tianjin China; ^2^ Tianjin Research Institute of Anesthesiology Tianjin China; ^3^ Department of Anesthesiology, Huashan Hospital Fudan University Shanghai China

**Keywords:** brain damage, hydrogen, phosphoproteomics, PI3K‐Art signalling pathway, sepsis

## Abstract

Our previous studies illustrated that 2% H_2_ inhalation can protect against sepsis‐associated encephalopathy (SAE) which is characterized by high mortality and has no effective treatment. To investigate the underlying role of protein phosphorylation in SAE and H_2_ treatment, a mouse model of sepsis was constructed by caecal ligation and puncture (CLP), then treated with H_2_ (CLP + H_2_). Brain tissues of the mice were collected to be analysed with tandem mass tag‐based quantitative proteomics coupled with IMAC enrichment of phosphopeptides and LC–MS/MS analysis. In proteomics and phosphoproteomics analysis, 268 differentially phosphorylated proteins (DPPs) showed a change in the phosphorylated form in the CLP + H_2_ group (*p* < 0.05). Gene ontology analysis revealed that these DPPs were enriched in multiple cellular components, biological processes, and molecular functions. KEGG pathway analysis revealed that they were enriched in glutamatergic synapses, tight junctions, the PI3K‐Akt signalling pathway, the HIF‐1 signalling pathway, the cGMP‐PKG signalling pathway, the Rap1 signalling pathway, and the vascular smooth muscle contraction. The phosphorylated forms of six DPPs, including ribosomal protein S6 (Rps6), tyrosine 3‐monooxygenase/tryptophan 5‐monooxygenase activation protein gamma (Ywhag/14–3‐3), phosphatase and tensin homologue deleted on chromosome ten (Pten), membrane‐associated guanylate kinase 1 (Magi1), mTOR, and protein kinase N2 (Pkn2), were upregulated and participated in the PI3K‐Akt signalling pathway. The WB results showed that the phosphorylation levels of Rps6, Ywhag, Pten, Magi1, mTOR, and Pkn2 were increased. The DPPs and phosphorylation‐mediated molecular network alterations in H_2_‐treated CLP mice may elucidate the biological roles of protein phosphorylation in the therapeutic mechanism of H_2_ treatment against SAE.

## 1 INTRODUCTION

Sepsis, which is caused by a series of pathophysiological processes, such as the excessive release of inflammatory factors and an imbalance between anti‐inflammatory and proinflammatory responses, leads to multiple organ dysfunction syndrome (MODS)^1^ and is the primary reason for admission into the intensive care unit.^2^, ^3^ Among the types of MODS caused by sepsis, sepsis‐associated encephalopathy (SAE) is the most serious and has high morbidity^4^ and mortality rates because the central nervous system is one of the most vulnerable organs.^5^

As the most widespread gas in the universe, hydrogen gas (H_2_) has been proven to alleviate many diseases and exert anti‐inflammatory, anti‐apoptotic, and antioxidant effects.^6^ Our previous studies have demonstrated that inhalation of 2% H_2_ can alleviate sepsis and protect against sepsis‐induced injury to various organs, such as the lungs, intestines, and brain.^7^, ^8^, ^9^ However, the therapeutic mechanism of H_2_ is still unknown.

With the widespread application and continuous development of molecular‐based technologies, proteomics and phosphoproteomics analysis have been used in various studies to explore disease mechanisms and therapeutic strategies.^10^ Phosphorylation and dephosphorylation are regulated by kinases and phosphatases, respectively. Approximately 2%–5% of genes in the human genome encode protein kinases and phosphatases.^11^ Previously, our studies demonstrated the differences in protein levels in lung tissues and intestinal tissues from septic animals and those from normal animals.^12^, ^13^

In this study, tandem mass tag (TMT)‐IMAC‐LC–MS/MS was used to investigate the differences in the levels of phosphoproteins and changes in phosphorylation‐mediated signalling pathways in the brain tissues of septic mice treated with H_2_ compared with those of untreated septic mice to clarify the specific mechanism underlying the therapeutic effect of H_2_ on sepsis.

## 2 MATERIALS AND METHODS

### 2.1 Animals and experiments

We purchased healthy adult male C57BL/6J mice (weighing 23–26 g and aged 8 weeks) from the Experimental Animal Center of the Academy of Military Sciences. The mice had free access to food and water and were housed in cages in a stable environment (temperature, 22–25°C; humidity, 55% ± 10%; and 12:12 h light–dark cycle). In this study, a total of 80 mice were randomly divided into four groups: the sham, sham + H_2_, caecal ligation and puncture (CLP), and CLP + H_2_ groups. The mice in the CLP and CLP + H_2_ groups underwent CLP to construct a sepsis model, while mice in the sham and sham + H_2_ groups underwent sham surgery. At 1 and 6 h following sham surgery or CLP, the mice in the sham + H_2_ and CLP + H_2_ groups were administered 2% H_2_ by inhalation for 60 min. The 7‐day survival rates of the mice after sham surgery or CLP were assessed (*n* = 20 per group). A total of 40 mice were used for brain proteomics and phosphoproteomics analysis (*n* = 10 mice per group), 24 mice were used for WB (*n* = 6 mice per group), and 16 mice were used for haematoxylin and eosin, Nissl staining, and TUNEL staining (*n* = 3 mice per group).

### 2.2 CLP model

CLP was performed as previously described.^9^ Before the operation, all the animals fasted for 8 h. The mice were anaesthetized by isoflurane inhalation and placed in a prone position. The skin was sterilized, a 1‐cm incision was made in the abdomen, the caecum was exposed, and 40% of it was ligated. Then, the caecum was punctured with a 21‐gauge needle, and approximately 0.3 ml of the caecal content was pushed out using sterile forceps. The abdominal muscles and skin were closed with simple running sutures and metallic clips, respectively, and then the caecum was returned to the abdominal cavity. After CLP or sham surgery, the mice were subcutaneously injected with saline solution (1 ml) and administered lidocaine cream (cat# H20063466) for pain relief. The postoperative mice were housed under controlled conditions (room temperature of 20–25°C), and a heating pad was used to prevent hypothermia.

### 2.3 H_2_ treatment

H_2_ treatment was applied as described in our previous study.^13^ Briefly, the mice were placed in a closed resin box with an inlet and an outlet. H_2_ was delivered using a TF‐1 gas flowmeter (4 L/min; YUTAKA Engineering Corp). To monitor the H_2_ concentration, a detector (HY‐ALERTA Handheld Detector Model 500; H2 Scan) was used. The CO_2_ expired by the mice was absorbed by Baralyme. The mice in the sham + H_2_ and CLP + H_2_ groups inhaled 2% H_2_ gas for 60 min, 1 h, or 6 h following CLP or sham surgery, while mice in the sham and CLP groups inhaled room air.

### 2.4 Haematoxylin and eosin staining

As described in a previous study,^14^ 24 h following sham surgery or CLP, the mice in each experimental group were deeply anaesthetized, and their brain tissues were collected for haematoxylin and eosin staining. The mice were transcardially perfused with phosphate‐buffered saline (PBS) followed by 4% paraformaldehyde in 0.1 M phosphate buffer (pH 7.4). The brain tissues were excised, fixed with 10% formalin, embedded in paraffin, and cut into sections. The samples were stained with haematoxylin and eosin following deparaffinization and dehydration. Regional neuropathological changes were observed using a BX51 microscope (Olympus).

### 2.5 Nissl staining

Brain tissues were cut into 10‐μm sections and stained with 1% crystal violet (Sigma) as described previously.^7^ Subsequently, pyramidal neurons in the hippocampal region were observed, and Nissl bodies in the hippocampal region were counted under a microscope.

### 2.6 TUNEL staining

Neuronal apoptosis was evaluated using the TUNEL assay 24 h after sham surgery or CLP. The nucleotide‐labelling mix (TUNEL reagent) contained fluorescein‐dUTP and fluorescein‐dNTPs, both of which are used for TUNEL staining for in situ analysis of apoptosis. In addition, a nucleotide‐labelling mix was used in combination with the TUNEL enzyme to prepare the TUNEL reaction mixture. Cells exhibiting DNA strand breaks were labelled with this reaction mixture, allowing us to analyse and quantify the degree of apoptotic cell death at the single‐cell level among cells and in tissues. Apoptotic cells were stained green, and the nuclei of all the cells were stained blue with DAPI.

### 2.7 Sample collection

First, 40 mice from all the experimental groups were anaesthetized 24 h following sham surgery or CLP. Subsequently, the heart was exposed, the right atrial appendage was cut, a cardiac puncture was performed using a sterile 18‐gauge needle, and the heart was perfused via the left ventricle with precooled PBS to remove the blood. Next, the brain tissue was collected after removing the skull, after which the sample was quickly frozen using liquid nitrogen and stored at −80°C for subsequent proteomics and phosphoproteomics analysis. To control for individual differences, six samples from each group were pooled. A total of three groups of six samples including 10 biological replicates and two technical replicates were subjected to iTRAQ‐based quantitative proteomics and phosphoproteomics analysis.

### 2.8 Protein extraction and digestion

Two hundred micrograms of protein from each sample were mixed with 30 μl SDT buffer (4% SDS, 100 mM DTT, and 100 mM Tris–HCl [pH 7.6]). The concentration of protein was quantified with the BCA Protein Assay Kit (Bio‐Rad). Trypsin protein digestion was performed according to the filter‐aided sample preparation procedure described by Matthias Mann.^15^ The digest peptides of each sample were desalted on C18 cartridges (Empore™ SPE Cartridges C18 [standard density], bed I.D. 7 mm, volume 3 ml, Sigma), concentrated by vacuum centrifugation and reconstituted in 40 μl of 0.1% (v/v) formic acid. The peptide content was estimated by UV light spectral density at 280 nm using an extinction coefficient of 1.1 of 0.1% (g/l) solution that was calculated based on the contents of tryptophan and tyrosine in vertebrate proteins.

### 2.9 TMT labelling and fractionation using the high‐pH reversed‐phase method

A 100 μg peptide mixture from each sample was labelled using TMT reagent (Thermo Fisher Scientific) according to the manufacturer's instructions. For fractionation using the high‐pH reversed‐phase method, a Pierce high pH reversed‐phase fractionation kit (Thermo Scientific) was used to fractionate the TMT‐labelled digest samples into six fractions by increasing acetonitrile step‐gradient elution according to the instructions.

### 2.10 Phosphopeptide enrichment and LC–MS/MS analysis

#### 2.10.1 IMAC enrichment method

Phosphopeptide enrichment was carried out using a High‐SelectTM Fe‐NTA Phosphopeptide Enrichment Kit (Thermo Scientific) according to the manufacturer's instructions. After lyophilization, the phosphopeptide peptides were resuspended in 20 μl loading buffer (0.1% formic acid).

#### 2.10.2 LC–MS/MS analysis

LC–MS/MS analysis was performed on a Q Exactive HF mass spectrometer (Thermo Scientific) coupled to the Easy nLC system (Proxeon Biosystems, now Thermo Fisher Scientific) for 120 min. The peptides were loaded onto a reverse‐phase trap column (Thermo Scientific Acclaim PepMap100, 100 μm*2 cm, nanoViper C18) connected to a C18 reversed‐phase analytical column (Thermo Scientific Easy Column, 10 cm long, 75 μm inner diameter, and 3 μm resin) in buffer A (0.1% formic acid) and separated with a linear gradient of buffer B (84% acetonitrile and 0.1% formic acid) at a flow rate of 300 nl/min controlled by IntelliFlow technology. The mass spectrometer was operated in positive ion mode. MS data were acquired dynamically using a data‐dependent top 10 method by choosing the most abundant precursor ions from the survey scan (300–1800 m/z) for HCD fragmentation. The automatic gain control (AGC) target was set to 3e6, the maximum injection time was set to 10 ms, and the dynamic exclusion duration was set to 40.0 s. Survey scans were acquired at a resolution of 70,000 at m/z 200, the resolution for HCD spectra was set to 17,500 at m/z 200, and the isolation width was set to 2 m/z. The normalized collision energy was set to 30 eV, and the underfill ratio, which specifies the minimum percentage of the target value likely to be reached at maximum fill time, was set to 0.1%. The instrument was run with peptide recognition mode enabled.

### 2.11 Protein identification and quantification

MS/MS spectra were searched using the MASCOT engine (Matrix Science; version 2.2) embedded into Proteome Discoverer 2.4. The following parameters were used: enzyme: trypsin; max missed cleavages: 2; fixed modifications: carbamidomethyl (C), iTRAQ4/8 plex (N‐term), iTRAQ 4/8 plex (K) or TMT 6/10/16 plex (N‐term), iTRAQ6/10/16 plex (K); variable modifications: oxidation (M)/phospho(ST)/phospho(Y); database: Swissprot_Mus_musculus_17063_20210106.fasta; peptide mass tolerance: ±20 ppm; fragment mass tolerance: 0.1 Da; peptide FDR: ≤0.01; and protein FDR: ≤0.01.

### 2.12 Bioinformatics analysis

#### 2.12.1 Cluster analysis

First, quantitative target protein data were normalized to the interval (−1,1). Then, the Complexheatmap R package (R Version 3.4) was used to classify both samples and protein expression levels (distance algorithm: Euclid; connection mode: average linkage) and generate a hierarchical clustering heatmap.

#### 2.12.2 GO enrichment and KEGG pathway analysis

For gene ontology (GO) enrichment analysis, the protein sequences of the selected differentially expressed proteins were locally searched using NCBI BLAST+ client software (ncbi‐blast‐2.2.28+‐win32.exe) and InterProScan to find homologous sequences. Then, the GO terms were mapped, and the sequences were annotated using the software program Blast2GO. The GO enrichment analysis results were plotted by R scripts. KEGG pathway analysis of the target protein set was performed using the KEGG Automatic Annotation Server (KAAS).

#### 2.12.3 Enrichment analysis

Enrichment analysis was performed based on Fisher's exact test considering all quantified proteins as a background dataset. The Benjamini–Hochberg correction for multiple testing was further applied to adjust the derived *p* values. Only functional categories and pathways with *p* values <0.05 were considered significantly enriched.

### 2.13 Western Blot

Twenty‐four hours after CLP or sham surgery, brain tissues were collected from the mice, weighed, and lysed using RIPA buffer (Solarbio) containing protease inhibitors (Solarbio) to extract total protein. The extracted protein samples were used to verify the expression levels of the following proteins: Rps6, Ywhag/14‐3‐3, Pten, Magi1, mTOR, and Pkn2. The total protein concentration was measured using a BCA protein assay kit (Thermo Fisher Scientific). Loading buffer (Beijing Solarbio Science & Technology Co., Ltd.) was added to each sample, and the samples were boiled, denatured, and stored at −80°C. Proteins were separated according to molecular weight using SDS–PAGE, and the proteins were electrically transferred to a polyvinylidene fluoride membrane (Millipore). The membrane was blocked using 5% skim milk powder in Tris/Tween‐20 for 2 h and then incubated with the following primary antibodies at 4°C overnight: Rps6 (1:1000, cat# ab225676, Abcam), Ywhag/14‐3‐3 (1:500, cat# DF3493, Affinity), PTEN (1:1000, cat# ab267787, Abcam), Magi1 (1:500, cat# 55048‐1‐AP, Proteintech), mTOR (1:10000, cat# ab134903, Abcam), Pkn2 (1:1000, cat# ab138514, Abcam), phosphorylated Rps6 (S240 + S244) (1:5000, cat# ab215214, Abcam), phosphorylated PTEN (1:500, cat# DF3493, Affinity), phosphorylated mTOR (S2448) (1:1000, cat# ab109268, Abcam), phosphorylated Pkn2 (T816) (1:1000, cat# ab124709, Abcam), and β‐actin (1: 5000, Proteintech). The membrane was incubated with goat anti‐rabbit or anti‐mouse secondary antibodies (1:5000, KPL) for 1 h at room temperature and then washed five times with Tris‐buffered saline/Tween‐20. The protein bands were visualized with enhanced chemiluminescence reagents. The density of each protein band was quantified using ImageJ software, and the relative levels of Rps6, Ywhag, PTEN, Magi1, mTOR, and Pkn2 were normalized to the level of β‐actin. The experiment was repeated six times.

### 2.14 Statistical analysis

Statistical analysis was performed with GraphPad Prism software (version 8.0) and spss statistical software (version 21.0). Survival rates are reported as percentages, and analysis was performed by the log‐rank test. The data are presented as the mean ± standard deviation. Two‐way repeated‐measures anova followed Tukey's multiple comparisons test was used to analyse differences between the treatment groups. Unpaired *t*‐test (if the values were normally distributed) or the Mann–Whitney test (if the values were nonnormally distributed) was employed to analyse differences between the two groups. For all tests, *p* < 0.05 was considered statistically significant.

## 3 RESULTS

### 3.1 Survival rate

**FIGURE 1:**
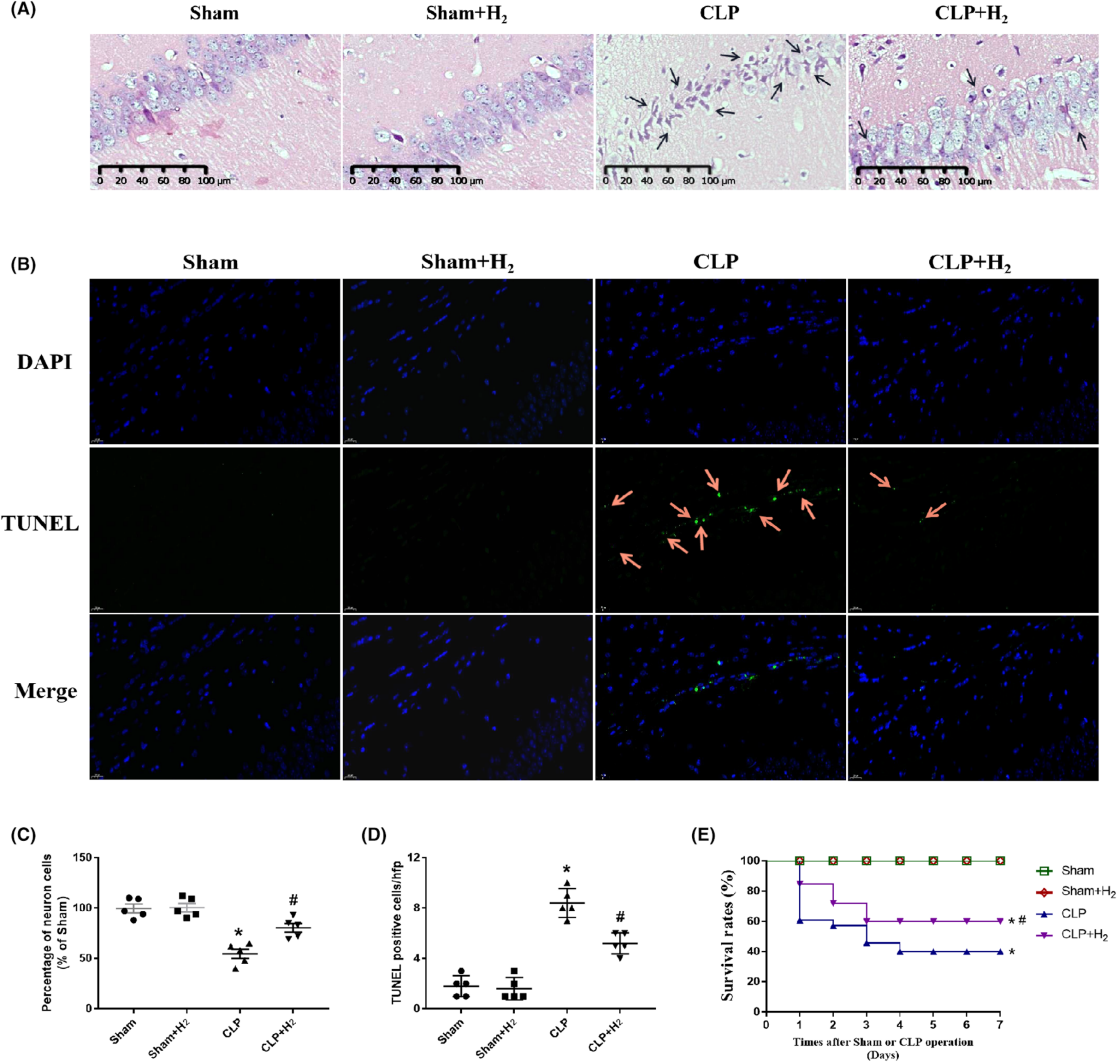
H_2_ can relieve the hippocampal histopathology damage and improve the 7‐day survival rates in septic mice. At 24 h following sham or CLP operation, (A) haematoxylin and eosin staining was used to detect the hippocampal histopathology changes. Regular morphology of the pyramidal neurons in the hippocampus was observed in the sham and sham + H_2_ groups. There were a large number of damaged neurons (indicated using black arrows) in the CLP group, and a significantly decreased number following H2 treatment (C). Neuronal apoptosis was detected by TUNEL staining (B); and the TUNEL‐positive cells per high‐powered field are counted (D). *n* = 5 per group. (E). The survival rates of mice were recorded for 7 days (*n* = 20). *p < 0.05 vs. Sham group, #*p* < 0.05 vs. CLP group. CLP, cecal ligation and puncture; H_2_, hydrogen

After sham surgery or CLP, several physiological and pathological changes, including unkempt hair, loose stools, a reduction in activity, and an increase in respiratory rate, were observed, consistent with a previous study.^13^ All mice in the sham and sham + H_2_ groups survived to 7 days, while the number of surviving mice at 7 days was significantly reduced in the sepsis and sepsis + H_2_ groups (*p* < 0.05). Nevertheless, the survival rate in the sepsis + H_2_ group was significantly improved compared with that in the sepsis group (40% vs. 60%, *p* < 0.05) (Figure 1E).

### 3.2 Detrimental histological changes in the hippocampi of septic mice

Haematoxylin and eosin staining showed that in the sham and sham + H_2_ groups, pyramidal neurons in the hippocampal area had a clear structure without obvious abnormalities (Figure 1A) and that there was no significant difference in pyramidal neuron structure between these two groups (Figure 1C, *p* > 0.05). However, in the CLP group, pyramidal neurons were seriously damaged and disorderly arranged, the cytoplasm was deeply stained, and nuclei were pyknotic (Figure 1A). Neuronal damage was alleviated in the CLP + H_2_ group compared with the CLP group, and most of the neurons in the CLP + H_2_ group showed a normal structure with slight damage. The number of normal neurons in the hippocampal area was significantly reduced in the CLP and CLP + H_2_ groups compared with the sham group; furthermore, there were significantly more normal neurons in the CLP + H_2_ group than in the CLP group (*p* < 0.05). Nissl staining (Figure S1) showed similar changes in the arrangement and structure of the pyramidal neurons as haematoxylin and eosin staining; the cells were sparsely arranged and an increased number of dissolved Nissl bodies were observed in the neuronal cytoplasm in the CLP and CLP + H_2_ groups compared with the sham group (Figure S2, *p* < 0.05). In addition, the results showed that the number of Nissl bodies was increased in the CLP + H_2_ group compared with the CLP group (*p* < 0.05) (Figure S2). The TUNEL staining results and quantification of TUNEL staining showed that there were fewer surviving neurons in the CLP group than in the sham group and that there were more TUNEL neurons in the CLP group than in the CLP + H_2_ group (Figure 1B,D).

### 3.3 Changes in protein phosphorylation in the brain tissues of septic mice

**FIGURE 2:**
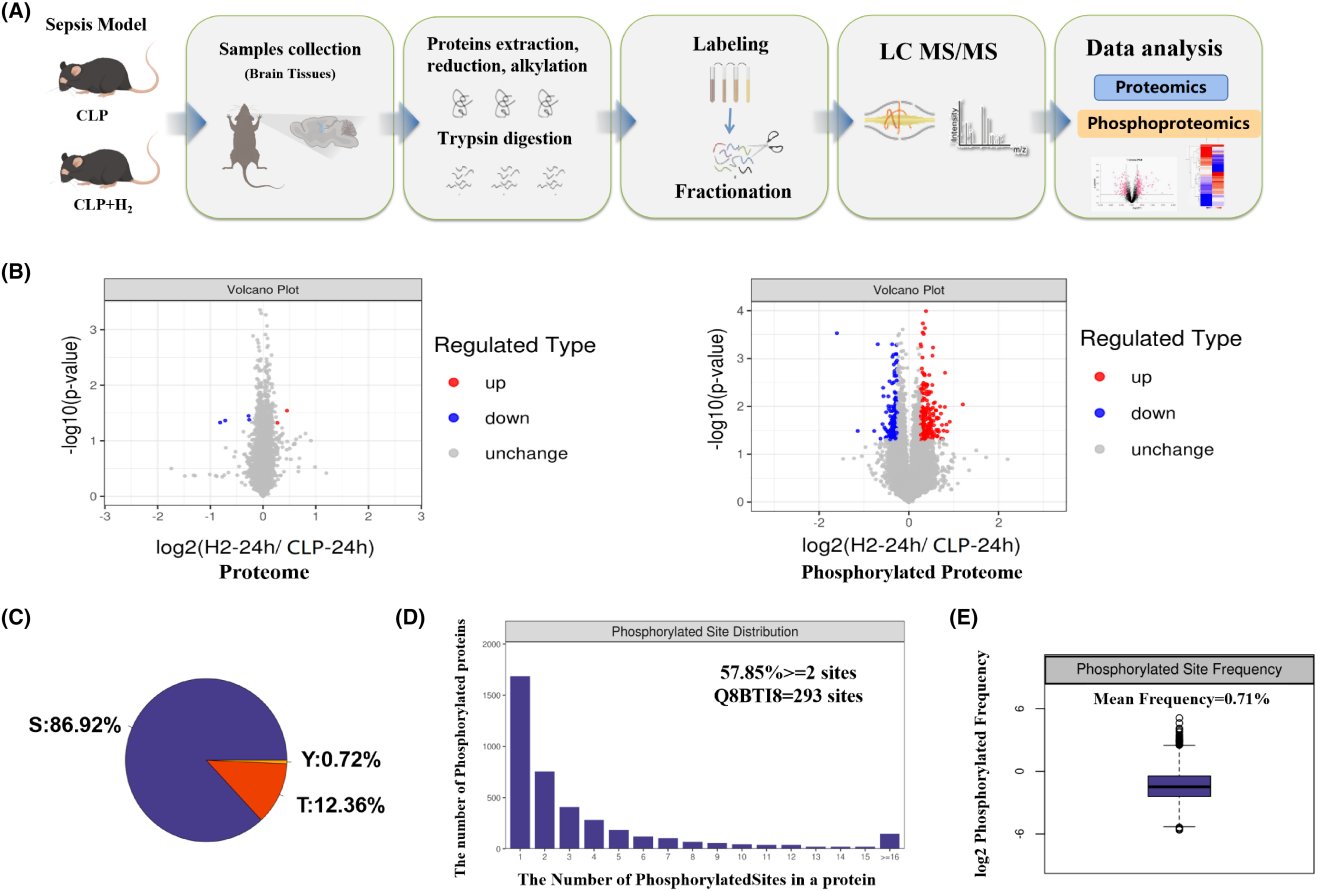
Identification and characterization of phosphoproteins in the brain tissues of septic mice treated with H_2_. (A) Experimental workflow to analyse the proteome and phosphoproteome of brain tissues derived from CLP and CLP + H_2_ mice. (B) Volcano plots showing the distribution of the significance and fold change of identified proteins and phosphopeptides between CLP + H_2_ mice and CLP mice. All data points are plotted in log2 (fold change) on the *x*‐axis and −log10 (*p*‐value) on the *y*‐axis. Blue dashed lines indicate a cutoff of fold change >1.2 or <0.05, one‐sample *t*‐test). (C) The proportion of Ser, Thr, and Tyr phosphorylation sites in identified phosphopeptides. (D) Histogram depicting the distribution of the number of phosphorylation sites per protein. (E) Frequency distribution of phosphorylation modification sites

To determine if the changes in protein phosphorylation identified by proteomics analysis are associated with the therapeutic effects of H_2_ on brain damage in septic mice, we collected brain tissues from CLP and CLP + H_2_ group mice for TMT (Thermo Fisher Scientific) or iTRAQ (Applied Biosystems) labelling. Then, we compared the changes in protein phosphorylation in three pairs of CLP + H_2_ group mice and CLP group mice using quantitative phosphoproteomics (Figure 2A).

A total of 4159 phosphoproteins, 12,237 phosphopeptides and 15,876 phosphosites with 12,228 quantifiable phosphorylated peptides and 15,864 quantifiable phosphorylated sites on 4155 modified proteins were identified. We further screened proteins and peptides with a fold change (FC) >1.2 and *p* value < 0.05 (*t*‐test) to identify modified peptides that showed a significant change in abundance. The levels of 223 and 184 phosphopeptides were increased and decreased, respectively in the CLP + H_2_ group compared to the CLP group. The magnitude and significance of the changes in protein phosphorylation levels between CLP and CLP + H_2_ group mice are presented in volcano plots (Figure 2B). The top 20 proteins with significant changes in phosphorylation levels are listed in Table 1. A total of 13,789 serine phosphorylation sites were identified in the brains of CLP + H_2_ group mice (accounting for 86.92% of total phosphopeptides), and other phosphorylation sites included threonine (1961) and tyrosine (114) phosphorylation sites (accounting for 12.36% and 0.72% of total phosphopeptides, respectively) (Figure 2C). In addition, most of the phosphoproteins (57.85%) contained more than two phosphorylation sites; for example, the Q8BTI8 protein had 293 phosphorylation sites (Figure 2D). The mean phosphorylation frequency of all quantified proteins was as high as 0.71 per hundred amino acids (Figure 2 E). These findings suggest that a prominent change in protein phosphorylation, mainly serine phosphorylation, is critically important for the therapeutic effect of H_2_ on brain damage in septic mice.

**TABLE 1:** Top 20 significant differences analysed phosphorylated proteins in the brain of CLP + H2 mice compared to CLP mice

Sequence	Protein group accessions	Protein names	Gene names	Fold change (CLP + H_2_/CLP)	*t*‐Test *p*‐value
[M].GKGGNQGEGSTER.[Q]	Q9Z0R9	Acyl‐CoA 6‐desaturase	Fads2	2.303221652	0.009094447
[R].ASTFCGTPDYIAPEILQGLK.[Y]	P28867	Protein kinase C delta type	Prkcd	1.880045652	0.020940324
[K].LDSFTQVFANQNLR.[I]	Q8BXJ2	Transcriptional‐regulating factor 1	Trerf1	1.806585741	0.033332229
[R].GGGSGGGDESEGEEVDED.[−]	O35295	Transcriptional activator protein Pur‐beta	Purb	1.788404595	0.024065562
[K].EGGGDSSASSPTEEEQEQGEMSACSDEGTAQEGK.[A]	P28667	MARCKS‐related protein	Marcksl1	1.749025465	0.001982334
[K].TSDSKEEIKEPESFNAAAQEAEAPYISIACDLIK.[E]	Q99P72	Reticulon‐4	Rtn4	1.745293381	0.010286863
[R].TSTFCGTPEFLAPEVLTETSYTR.[A]	Q8BWW9	Serine/threonine‐protein kinase N2	Pkn2	1.710356309	0.032159285
[K].GNDISSGTVLSDYVGSGPPSGTGLHR.[Y]	P70296	Phosphatidylethanolamine‐binding protein 1	Pebp1	1.692915353	0.047902314
[K].NLIDSMDQEAFHGFSFVNPK.[F]	P28867	Protein kinase C delta type	Prkcd	1.668933842	0.024541989
[R].STEASPSRDASPVGLK.[I]	Q9JJV4	Voltage‐dependent calcium channel gamma‐4 subunit	Cacng4	1.648650677	0.047080208
[R].VVETQWDVSSAASPESPEECARPEEPASPEDPPSR.[H]	Q8BNW9	Kelch repeat and BTB domain‐containing protein 11	Kbtbd11	1.640615265	0.014203907
[K].EGDTEEEAGPQAAEPSTPSGPESGPTPASAEQNE.[−]	P28667	MARCKS‐related protein	Marcksl1	1.622521951	0.010076547
[K].APSPETQTCILDTDGRGSPDDSKEK.[S]	Q7TSN6	G‐protein coupled receptor 151 protein	Gpr151	1.589256681	0.033903225
[R].AELPPPYTAIASPGTSGIPVINCR.[V]	Q9CZX7	Type 2 phosphatidylinositol 4,5‐bisphosphate 4‐phosphatase	Pip4p2	1.582843397	0.017605492
[R].TFQQIQEEEDDDYPGSYSPQDPSAGPLLTEELIK.[A]	Q9CSU0	Regulation of nuclear pre‐mRNA domain‐containing protein 1B	Rprd1b	1.573534368	0.030856321
[R].TEATQGLDYVPSAGTISPTSSLEEDKGFK.[S]	Q9QYR6	Microtubule‐associated protein 1A	Map1a	1.55597753	0.041539364
[K].ESESDQEPEEEIGMTSEKNDETESTETSVLK.[S]	Q8C8R3	Ankyrin‐2	Ank2	1.548904563	0.032890481
[R].RPGSHPSSHR.[S]	Q9QYB2	Dachshund homologue 1	Dach1	1.546699184	0.042744862
[K].ETEVSKGSAESPDEGITTTEGEGECEQTPEELEPVEK.[Q]	P14873	Microtubule‐associated protein 1B	Map1b	1.545098188	0.018198383
[K].VDYLTPDFPSLSYPNYYTLMTGR.[H]	Q8BGN3	Glycerophosphocholine cholinephosphodiesterase ENPP6	Enpp6	1.541936352	0.017125863

### 3.4 Combined proteomics and phosphoproteomics analysis

**FIGURE 3:**
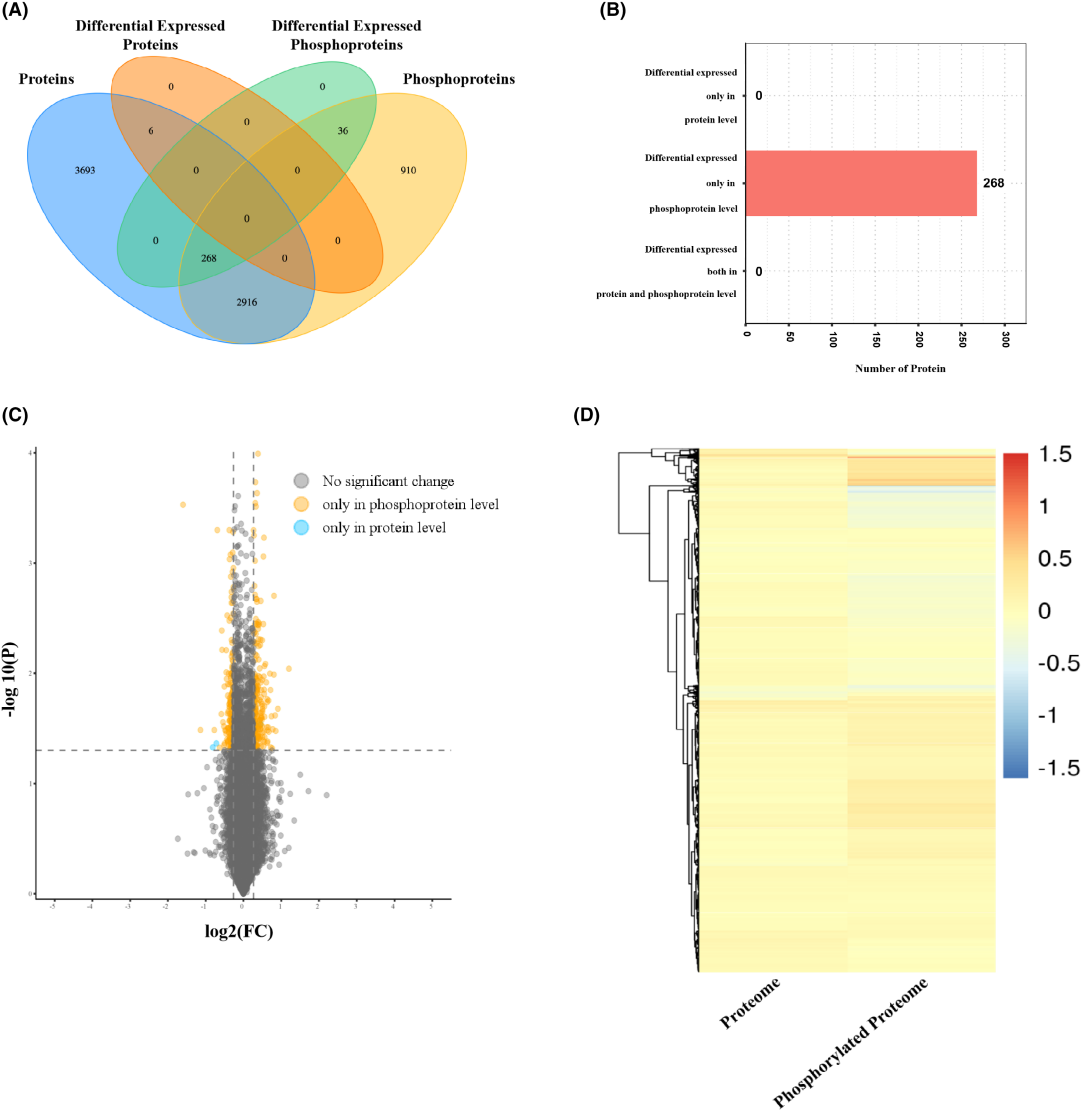
Combination of proteomics and phosphorylated proteomics analysis. (A) and (B) A total of 6883 differentially expressed proteins in Multiple omics analysis in CLP + H_2_ mice vs. CLP mice and the 268 correlated proteins were differentially expressed only in phosphoproteins. (C) Volcano plot and (D) hierarchical clustering showing the distribution of the significance and fold change of identified phosphoproteins between CLP + H_2_ mice and CLP mice. All data points are plotted in log2 (fold change) on the *x*‐axis and −log10 (*p*‐value) on the *y*‐axis.

Proteins are the functional executors of life activities, and their regulatory function is mainly affected by their expression levels and posttranslational modifications (PTMs). They ultimately affect the phenotypic changes of organisms. Previous studies have shown that H_2_ treatment leads to changes in protein levels and modification in the brain tissues of septic mice.^16^ Therefore, we performed a combined analysis of proteins and modified proteins to further assess the ability of H_2_ to alter protein function in mice with brain injury. As shown in Figure 3A–C, the levels of 6883 proteins were quantified and six showed differential abundance. Moreover, the levels of 4130 phosphoproteins were quantified, and 304 showed differential abundance. Among the 3184 differentially abundance proteins, 268 showed a change in the abundance of the phosphorylated form, whereas none showed a change in the abundance of total protein or the abundance of both total protein and the phosphorylated form (Figure 3D). The top 20 differentially abundant proteins that showed a change in the abundance of the phosphorylated form in the brains of CLP + H_2_ group mice compared to those of CLP group mice are listed in Table 2.

**TABLE 2:** Top 20 correlated proteins differential expressed only in phosphoproteins in the brain of CLP + H_2_ mice compared to CLP mice

Protein ID	Protein name	Gene name	Phosphorylated site	Amino acid	Phosphorylated peptide FC(CLP + H_2_ vs. CLP)	*p*‐Value
Q9QXL2	Kinesin‐like protein KIF21A	Kif21a	1273	S	2.837410745	0.083060323
Q9DCU2	Plasmolipin	Pllp	12–34	T/S	2.538418215	0.21685138
Q8CGK7	Guanine nucleotide‐binding protein G(olf) subunit alpha	Gnal	192	S	2.358050516	0.124072195
Q9Z0R9	Acyl‐CoA 6‐desaturase	Fads2	11	S	2.303221652	0.009094447
P20357	Microtubule‐associated protein 2	Map2	1478–1488	S	2.131415893	0.408581011
P22005	Proenkephalin‐A	Penk	171	S	1.984746754	0.098840197
P08553	Neurofilament medium polypeptide	Nefm	550	S	1.974892644	0.146378466
Q99P72	Reticulon‐4	Rtn4	163–189	S/T	1.95803817	0.196019669
O54781	SRSF protein kinase 2	Srpk2	443–482	S462	1.923084144	0.544756222
O54781	SRSF protein kinase 2	Srpk2	443–482	S465	1.923084144	0.544756222
O54781	SRSF protein kinase 2	Srpk2	477	S	1.923084144	0.544756222
O54781	SRSF protein kinase 2	Srpk2	443–482	S452	1.923084144	0.544756222
P08553	Neurofilament medium polypeptide	Nefm	715	S	1.918801149	0.13835512
P28867	Protein kinase C delta type	Prkcd	505	T	1.880045652	0.020940324
Q8C0T5	Signal‐induced proliferation‐associated 1‐like protein 1	Sipa1l1	1421–1450	S/T	1.878711943	0.195293277
P83510	Traf2 and NCK‐interacting protein kinase	Tnik	696	T	1.868967251	0.195683939
P83510	Traf2 and NCK‐interacting protein kinase	Tnik	696–728	S702	1.868967251	0.195683939
P83510	Traf2 and NCK‐interacting protein kinase	Tnik	696–728	S700	1.868967251	0.195683939
P83510	Traf2 and NCK‐interacting protein kinase	Tnik	696–728	S703	1.868967251	0.195683939
P83510	Traf2 and NCK‐interacting protein kinase	Tnik	696–728	S701	1.868967251	0.195683939

### 3.5 Classification of proteins and phosphorylated proteins into functional groups

**FIGURE 4:**
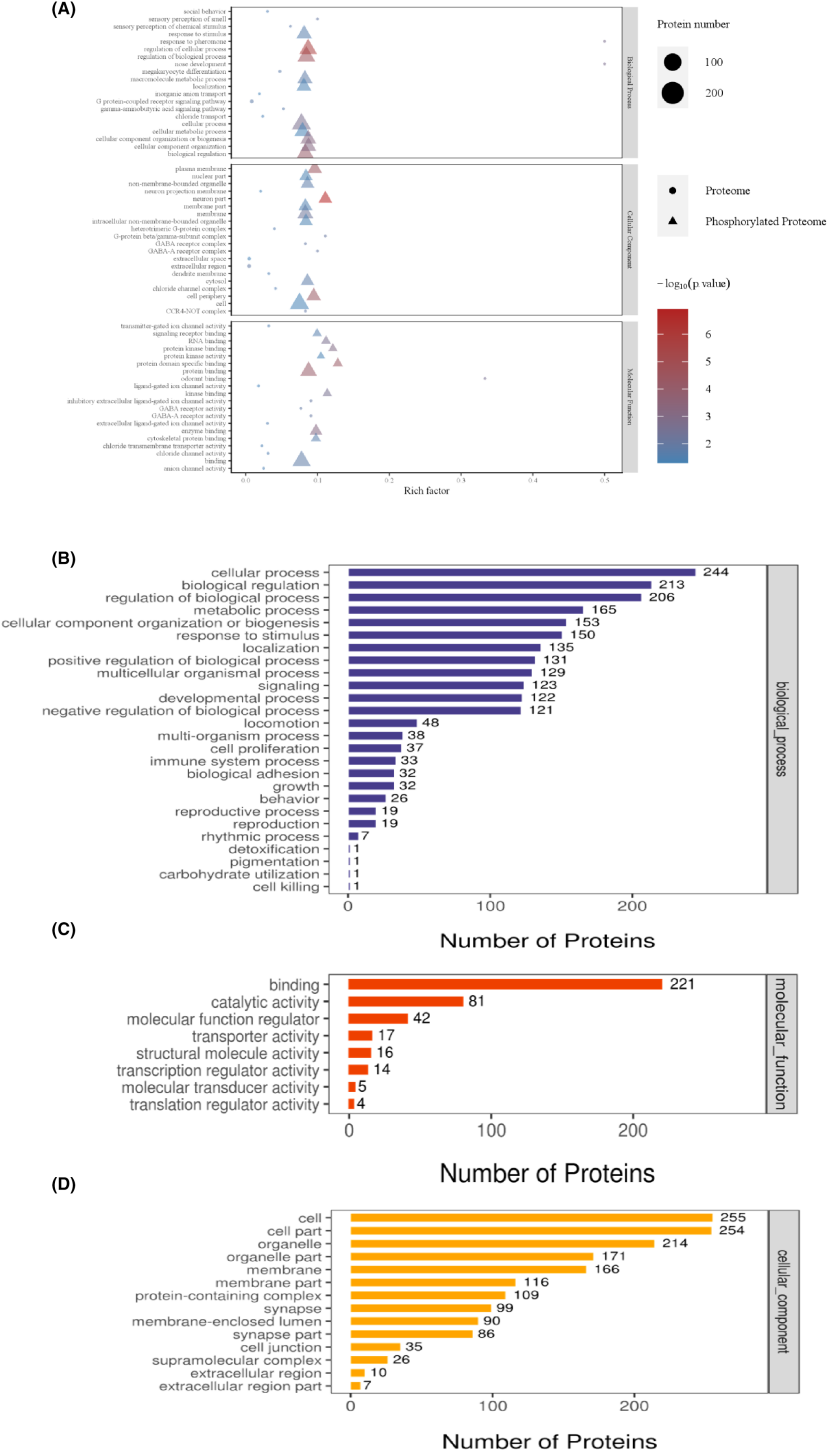
Functional classification of differentially expressed proteins and phosphoproteins. The subcellular and functional categories of 268 upregulated phosphoproteins (fold change >1.2 or <0.05, one‐sample *t*‐test) are based on the gene ontology annotations using the ANTHER Classification System for the following categories: Comparison diagram of GO functional enrichment at single omics level (A), biological process (B), molecular function (C), and cellular component (D)

Changes in the levels or phosphorylation of proteins are implicated in the regulation of biological functions. To elucidate the synergistic relationship between the abundance of modification of proteins and how they are regulated, the differentially abundant proteins in the brains of CLP + H_2_ group mice (FC >1.2 and *p* < 0.05, CLP + H_2_ group vs. CLP group, *p* < 0.05, one‐sample *t*‐test) that were identified by proteomics and phosphoproteomics analysis were subjected to GO enrichment analysis (Figure 4A). A large number of the differentially abundant proteins were associated with cellular processes, including the regulation of biological processes (BPs), metabolic processes, and cellular organization and biogenesis (Figure 4B). We classified the differentially phosphorylated proteins (DPPs) into different functional groups and found that most are involved in binding and catalytic activity (Figure 4C). This proves the hypothesis that phosphorylation is a vital PTM that regulates protein interactions and the activity of biological molecules and related signalling cascades. Moreover, cellular component (CC) enrichment analysis revealed that the DPPs were enriched in organelles, membranes, and protein‐containing complexes (Figure 4D). These findings collectively suggest that the changes protein phosphorylation in CLP + H_2_ group mice may have contributed to the improvement of neuronal functions through various processes, including morphological reorganization, gene and protein expression, and metabolism.

### 3.6 Pathway analysis of the DPPs

**FIGURE 5:**
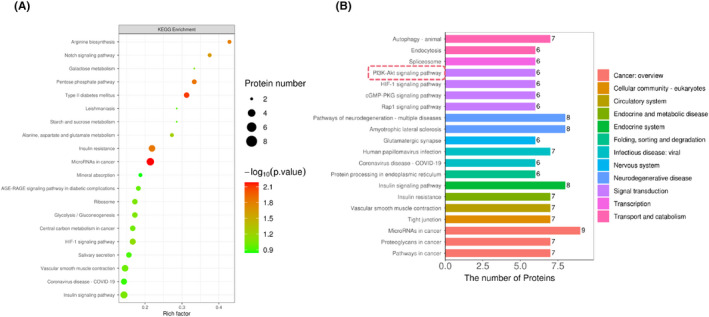
KEGG pathway analysis of the differentially expressed correlated Phosphoproteins. Differentially expressed phosphoproteins (CLP + H_2_ vs. CLP mice, *p* < 0.05, one‐sample *t*‐test) were subjected to DAVID online pathway analysis. (A)The KEGG pathway enrichment analysis of proteomics and phosphorylomic is shown. (B)The top 20 KEGG pathways are listed (FDR < 0.05). The *X*‐axis indicates the number of proteins, with the corresponding KEGG pathway marked on the *y*‐axis.

To further elucidate the biological roles of the DPPs (proteins for which only the phosphorylated form showed a change in abundance) and the signalling events they regulate, we performed pathway analysis of the 268 DPPs (*p* < 0.05, CLP + H_2_ group vs. CLP group, one‐sample *t*‐test) using the KAAS. KEGG pathway analysis of the differentially abundant proteins and DPPs was performed. The results are shown in a single image because they can be intuitively compared to identify synergistic relationships between protein expression and modification and differential regulation of these processes from the perspective of pathways (Figure 5A). We defined significant enrichment as an FDR <0.05. The top 20 pathways are shown in Figure 5B. The enriched KEGG pathways (i.e., glutamatergic synapse, amyotrophic lateral sclerosis, pathways of neurodegeneration‐multiple diseases) are related to the nervous system and neurodegenerative diseases. Glutamatergic synapses have been proven to be involved in neurodegenerative diseases; for example, glutamatergic receptors, such as n‐methyl‐d‐aspartate (NMDA) receptors, which are tightly modulated via protein phosphorylation, are found at glutamatergic synapses, and hyperactivation of NMDA receptors leads to excitotoxicity, synaptic failure, and ultimately neuronal death in Alzheimer disease (AD).^17^ In addition, the results showed that the DPPs in CLP + H_2_ group mice are closely associated with signal transduction pathways, including the PI3K‐Akt signalling pathway, HIF‐1 signalling pathway, cGMP‐PKG signalling pathway and Rap1 signalling pathway (Figure 5B). In particular, the PI3K‐Akt signalling pathway can be activated by external stimuli, resulting in the phosphorylation of the downstream signalling molecule AKT followed by the phosphorylation of the downstream signalling molecule mammalian target of rapamycin (mTOR), one of the differentially abundant phosphoproteins identified in our study. In this way, the PI3K‐Akt signalling pathway can regulate a wide variety of biological processes, including inflammation, cellular proliferation, autophagy, and apoptosis.^18^ Therefore, the PI3K‐Akt signalling pathway and up‐ and downstream signalling molecules may play a key role in the therapeutic effect of H_2_ on brain damage in septic mice.

### 3.7 Verification of PI3K‐Akt signalling pathway‐related DPPs in CLP + H_2_ group mice compared to CLP group mice

**FIGURE 6:**
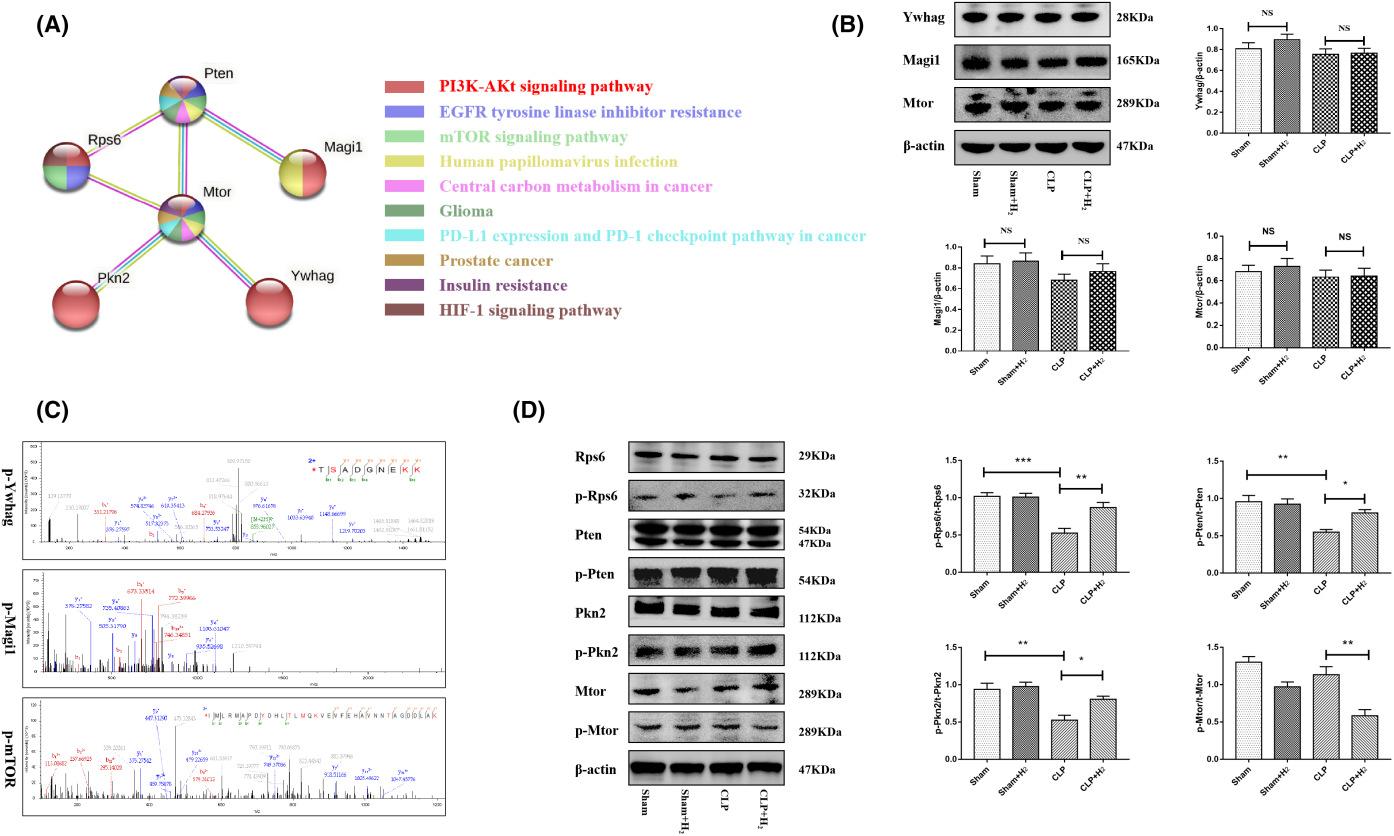
Differentially expressed phosphoproteins (DPPs) (Rps6, Pten, Ywhag, mTOR, Magi1, and Pkn2) participate in the PI3K‐AKT signalling pathway were significantly different in the therapeutic mechanism of hydrogen in CLP‐induced mice. (A) The western blot were used to identify the differentially expressed phosphoproteins and the total proteins. The results showed there were no changes in the total level of Ywhag, mTOR, and Magi1 quantified by the ratio of band density to those of β‐actin, respectively (B) and the MS/MS spectrums from Phosphorylated peptides. (C) (p‐Ywhag assigned to TSADGNEKK; p‐Magi1 assigned to AENEVPSPASSHHSSNQPASLTEEK; p‐mTOR assigned to IMLRMAPDYDHLTLMQKVEVFEHAVNNTAGDDLAK). The b and y ions including loss of ammonia and water were considered when we calculated the PTM score.) (D) The expression of Rps6, p‐Rps6, Pten, p‐Pten, mTOR, p‐mTOR, Pkn2, and p‐Pkn2 in CLP + H2 were detected by western blot. Quantitative analysis of p‐Rps6, p‐Pten, p‐mTOR, and p‐Pkn2 is shown as the ratio of band density to that of Rps6, Pten, mTOR, and Pkn2, respectively. NS, no significance; *p < 0.05; **p < 0.01; ***p < 0.001. Magi1, membrane‐associated guanylate kinase1; mTOR, mammalian target of rapamycin; Pkn2, protein kinase N2; Pten, phosphatase and tensin homologue deleted on chromosome ten; Rps6, Ribosomal protein S6; Ywhag/14–3‐3, tyrosine 3‐Monooxygenase/tryptophan 5‐monooxygenase activation protein gamma

Among the 268 DPPs in CLP + H_2_ group mice compared to CLP group mice identified by quantitative phosphoproteomics, we chose to validate the changes in the levels of ribosomal protein S6 (Rps6), tyrosine 3‐monooxygenase/tryptophan 5‐monooxygenase activation protein gamma (Ywhag), phosphatase and tensin homologue deleted on chromosome ten (Pten), protein kinase N2 (Pkn2), a membrane‐associated guanylate kinase 1 (Magi1), and mTOR, which are closely associated with the PI3K‐Akt signalling pathway. The protein–protein interaction network and associated pathways of these six proteins involved in the PI3K‐Akt signalling pathway are shown in Figure 6A. Immunoblotting showed that the total protein levels of Ywhag, Magi1, mTOR, Rps6, Pten, and Pkn2 were not different between the CLP + H_2_ group and CLP group (*p* > 0.05, Figure 6B,C). Mass spectrometry was then used to identify the differentially phosphorylated sites in Ywhag, Magi1, and mTOR, with the results are shown in Figure 6C. Western blotting (WB) showed that the levels of phosphorylated Rps6 (S240 + S244), Pten (S370), and Pkn2 (T816) were increased in septic mice treated with H_2_ compared with CLP model mice, while the total levels of those proteins were not changed (Figure 6D). These results are consistent with the results of MS/MS‐based quantitative phosphoproteomics in our study. In addition, consistent with a previous study,^19^ WB showed that the level of phosphorylated mTOR (S2448) was decreased in the CLP + H_2_ group compared with the CLP group. This was because H_2_ could regulate the mTOR‐autophagy signalling pathway to attenuate sepsis‐induced neuroinflammation.

In summary, in septic mice, H_2_ might regulate the phosphorylation of proteins (Rps6, Ywhag, Pten, Pkn2, Magi1, and mTOR) in the PI3K‐Akt signalling pathway to regulate cell metabolism, growth, and proliferation.

## 4 DISCUSSION

Sepsis is a systemic inflammatory syndrome that can lead to neurological impairment, including behavioural alterations and neurological impairment.^7^, ^12^ The CLP model, the gold standard model of sepsis, was applied in our present study.^20^ In our previous studies, we found that 2% H_2_ inhalation can alleviate sepsis and sepsis‐induced damage to organs, including the lung, liver, kidney, and brain.^7^, ^8^, ^9^, ^14^, ^21^ In this study, inhalation of 2% H_2_ effectively increased the 7‐day survival rate of septic mice. In our previous studies, we showed that the mechanism underlying the effect of H_2_ involves the regulation of apoptosis, the inflammatory response, and oxidative stress, possibly through the NK‐kB and Nrf2/HO‐1 signalling pathways.^6^ Furthermore, our research team previously performed proteomics analysis to assess the effect of H_2_ on sepsis and sepsis‐related damage to organs, including the lungs,^12^ intestine,^13^ and brain.^16^ In this study, we applied TMT‐IMAC‐LS–MS/MS to identify the changes in protein phosphorylated in H_2_‐treated CLP model mice and analysed proteomics and phosphoproteomics databases to determine the function of the DPPs and the pathways through which H_2_ exerts its therapeutic effect in CLP model mice. The results suggested that the PI3K‐Akt signalling pathway and phosphorylated proteins associated with this pathway are associated with the effect of H_2_.

Changes in protein expression generally results from mutations, splicing, and PTMs.^22^ PTMs, including phosphorylation, acetylation, ubiquitylation, nitration, and glycosylation, lead to proteomic variations and differences in protein function to a great extent. Protein phosphorylation plays a key role in almost every process in organisms. Phosphorylation is one of the most common PTMs; is involved in multiple complexes and crucial signalling pathways in eukaryotes; and regulates a wide range of basic cellular processes, including cell division, growth, and differentiation. Approximately one‐third of proteins are phosphorylated during the cell life cycle.^23^ To gain a deeper understanding of the phosphoproteomic profile of H_2_‐treated CLP model mice, TMT‐IMAC‐LC–MS/MS was used to identify phosphoproteins and phosphorylation sites and assess phosphorylation levels in the brain tissues of these mice relative to those of CLP model mice in this study. A total of 407 DPPs were identified, of which 223 showed a decrease in phosphorylation and 184 showed an increase in phosphorylation in the CLP + H_2_ group. In addition, serine phosphorylation sites (86.92%) accounted for a larger percentage of phosphorylation sites than threonine (12.36%) and tyrosine (0.72%) phosphorylation sites. To investigate the functions of the DPPs and their associated pathways, we analysed proteomics and phosphoproteomics databases and found that there were 268 DPPs in CLP + H_2_ group mice versus CLP group mice. BP GO enrichment analysis revealed that DPPs in the CLP + H_2_ group were mainly enriched in the regulation of cellular process and biological quality, indicating that H_2_ regulates apoptosis and autophagy in neurons, as previously reported.^19^, ^24^ KEGG pathway analysis showed that the DPPs were significantly enriched in 20 signalling pathways, including autophagy, endocytosis, the PI3K‐Akt signalling pathway, the HIF‐1 signalling pathway, the cGMP‐PKG signalling pathway, and the Rap1 signalling pathway, which are related to signal transduction, transport, and catabolism. Thus, the therapeutic effects of H_2_ may be associated with cell growth and metabolism.

It has been proven that sepsis regulates the PI3K‐Akt signalling pathway. The PI3K‐AktmTOR signalling pathway is a major regulator of cell growth and metabolism, promoting anabolic processes such as ribosome biogenesis and the synthesis of proteins, nucleotides, fatty acids, and lipids and inhibiting catabolic processes such as autophagy.^25^ Evidence has shown that dysregulation of mTOR signalling is linked to many human diseases, including diabetes, neurodegenerative diseases, and cancer.^26^ A previous study successfully showed that TG attenuates sepsis‐induced myocardial dysfunction by inhibiting myocardial autophagy by silencing PTEN expression and acting on the AKT/mTOR pathway.^27^ Based on the function of the DPPs and their associated signalling pathways, we selected Rps6, Ywhag, Pten, Pkn2, Magi1, and mTOR, which participate in the PI3K‐Akt signalling pathway, for verification. The total protein levels of Rps6, Ywhag, Pten, Pkn2, Magi1, and mTOR were not changed, but the phosphorylation levels of these proteins were increased. The WB results were consistent with the abovementioned data.

Other members of the PI3K‐Akt pathway that are upregulated in CLP + H_2_ group mice are also strongly associated with the therapeutic mechanism of H_2_. The expression of MAGI1, a cytoplasmic scaffolding protein, is decreased in some inflammatory diseases and in several cancers, including hepatocellular carcinoma and colorectal, cervical, breast, brain, and gastric cancers. MAGI1 appears to act as a tumour suppressor, modulating the activity of oncogenic pathways such as the PI3K/AKT and Wnt/β‐catenin pathways.^28^ Protein kinase N (PKN) is a member of the atypical protein kinase C subfamily and is known to regulate actin cytoskeleton rearrangement and cell migration. PKN may inhibit insulin signalling by directly interacting with PDK1 and Akt.^29^ A previous study showed that PKN2 contributes to cell adhesion‐mediated activation of Akt.^30^ Moreover, phosphoproteomics analysis of PKN2−/− mouse embryonic fibroblasts revealed that Akt pathway activity is increased in these cells.^31^ Furthermore, PKN2 knockdown impairs insulin‐responsive glucose metabolism and unexpectedly activates 5′‐adenosine monophosphate‐activated protein kinase, exerting downstream effects on lipid and protein metabolism.^32^ Evidence suggests that depletion of PRK1 and PRK2 in macrophages results in inflammasome‐dependent IL‐1β release, suggesting that these PRKs inhibit activation of the pyrin inflammasome (which forms in response to bacterial infection and induce interleukin [IL] release from macrophages to combat infection).^33^ Then, PRKs phosphorylate pyrin, leading to the recruitment of 14‐3‐3 proteins and inhibition of inflammasome activation. Genetic alterations that lead to dysregulation of PI3K signalling, most commonly PI3K mutations, HER2 amplification, and events that inactivate PTEN, are prevalent in cancer.^34^ Translational regulation of PTEN is a major process by which physiological PI3K signalling is homeostatically regulated and plays a role in reducing pathway activation by oncogenic PIK3CA mutants and the antitumour activity of PI3K pathway inhibitors.^35^ As an indirect downstream effector of mTOR, rpS6 not only participates in glucose homeostasis but is also essential for regulating the size of at least some cell types.^36^ In addition, it was recently shown that specific small inhibitory RNA‐mediated knockdown of RPS6 disrupts 40S ribosomal biogenesis, showing that RPS6 is a critical regulator of 5′ terminal oligopyrimidine (5′ TOP) translation.^37^

In our research, the activation of proteins related to the PI3K‐Akt signalling pathway (Rps6, Ywhag, Pten, Pkn2, Magi1, and mTOR) was closely associated with the therapeutic effect of H_2_ on brain damage in septic mice.

## 5 CONCLUSION

In the present study, we quantitatively assessed the changes in protein phosphorylation in the H_2_‐treated CLP model mice and identified functions and signalling cascades associated with the phosphoproteins that exhibited significant changes in abundance. Importantly, we revealed the critical involvement of the PI3K‐Akt signalling pathway and that related phosphoproteins (p‐Rps6, p‐Ywhag, p‐Pten, p‐Magi1, p‐mTOR, and p‐Pkn2) in the ability of H_2_ to relieve brain damage in septic mice. Taken together, these findings provide novel insights into the mechanisms underlying the therapeutic effect of H_2_ on sepsis and may offer a new therapeutic target for sepsis.

## AUTHOR CONTRIBUTIONS

**Yuanyuan Bai:** Conceptualization (lead); data curation (lead); formal analysis (equal); funding acquisition (equal); investigation (equal); methodology (equal); project administration (equal); resources (equal); software (equal); supervision (equal); validation (lead); visualization (equal); writing – original draft (lead); writing – review and editing (equal). **Li Li:** Data curation (equal); writing – original draft (equal). **Beibei Dong:** Data curation (equal); writing – original draft (equal). **Wanjie Ma:** Investigation (equal); software (equal). **Hongguang Chen:** Project administration (equal); supervision (equal). **Yu yonghao:** Funding acquisition (supporting); writing – review and editing (supporting).

## CONFLICT OF INTEREST

The authors declare that there are no conflicts of interest.

## Supporting information


FigureS1‐S2

## Data Availability

The data used to support the fndings of this study are available from the corresponding author upon request.
