# Supplementary material for: Phosphorylation‐mediated PI3K‐Art signalling pathway as a therapeutic mechanism in the hydrogen‐induced alleviation of brain injury in septic mice
Source: J Cell Mol Med. 2022 Oct 29;26(22):5713–27. doi: 10.1111/jcmm.17568 (PMC9667523; doi:10.1111/jcmm.17568)
Supplement: Supplementary file 1 — FigureS1‐S2 [file JCMM-26-5713-s001.docx]

# **Phosphorylation-Mediated PI3K-Art Signaling Pathway as a Therapeutic Mechanism in the Hydrogen-Induced Alleviation of Brain Injury in Septic Mice**

**List of Authors:**

Yuanyuan Bai^a,b,1^ (Baiyuanyuan0523@tmu.edu.cn)

Li Li^c,1^ (mazuilili@126.com)

Beibei Dong^a,b^(senyu1219@163.com)

Wanjie Ma^a,b^ [(mawanjie2021@tmu.edu.cn)](mailto:(15281356627@163.com))

Hongguang Chen^a,b^[(daguang521521@163.com](mailto:(daguang521521@163.com))

Yonghao Yu^a,b,*^ [(yyu@tmu.edu.cn)](mailto:(yyu@tmu.edu.cn))

^a^Department of Anesthesiology, Tianjin Institute of Anesthesiology, General Hospital of Tianjin Medical University, Tianjin, China, 300052

^b^Tianjin Research Institute of Anesthesiology, Tianjin, China,300052

^c^Department of Anesthesiology, Huashan Hospital, Fudan University, Shanghai, China, 200433

**Supplemental Figure 1**

**
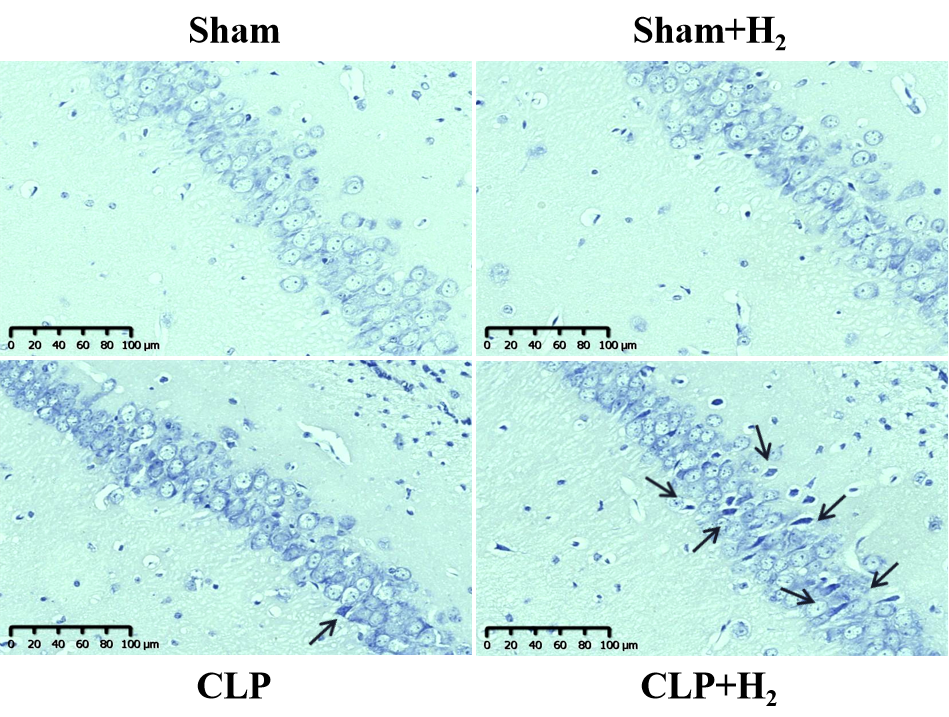
**

**Supplemental Figure 2**

**
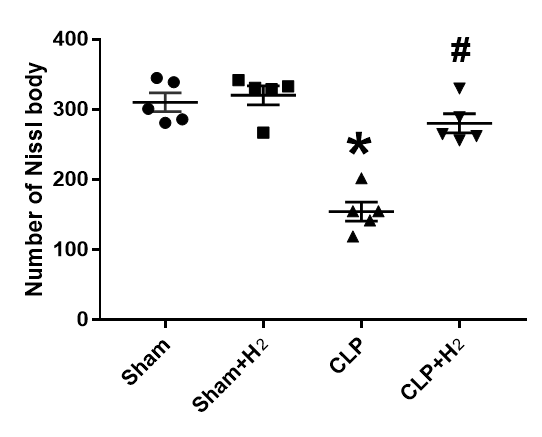
**

**Supplemental Figure 1 and 2** The Nissl was used to detect the hippocampal histopathology changes. The Nissl body, which was decreased or dissolved in CLP group, could be improved in CLP + H_2_ group. Scale bar, 100 μm. *P < 0.05 versus Sham group, #P < 0.05 versus CLP group. CLP, cecal ligation and puncture; H_2_, hydrogen.
